# Three Realizations and Comparison of Hardware for Piezoresistive Tactile Sensors

**DOI:** 10.3390/s110303249

**Published:** 2011-03-17

**Authors:** Fernando Vidal-Verdú, Óscar Oballe-Peinado, José A. Sánchez-Durán, Julián Castellanos-Ramos, Rafael Navas-González

**Affiliations:** Department of Electronics, University of Málaga, 29071 Málaga, Spain; E-Mails: oballe@uma.es (O.O.-P.); jsd@uma.es (J.A.S.-D.); jcramos@uma.es (J.C.-R.); rjnavas@uma.es (R.N.-G.)

**Keywords:** tactile sensors hardware, direct connection sensor-FPGA, PSoC

## Abstract

Tactile sensors are basically arrays of force sensors that are intended to emulate the skin in applications such as assistive robotics. Local electronics are usually implemented to reduce errors and interference caused by long wires. Realizations based on standard microcontrollers, Programmable Systems on Chip (PSoCs) and Field Programmable Gate Arrays (FPGAs) have been proposed by the authors for the case of piezoresistive tactile sensors. The solution employing FPGAs is especially relevant since their performance is closer to that of Application Specific Integrated Circuits (ASICs) than that of the other devices. This paper presents an implementation of such an idea for a specific sensor. For the purpose of comparison, the circuitry based on the other devices is also made for the same sensor. This paper discusses the implementation issues, provides details regarding the design of the hardware based on the three devices and compares them.

## 1. Introduction

Tactile sensors can be based on different principles, including capacitive, resistive or optical methods, and are oriented to a broad range of applications, for instance in assistive or industrial robotics or rehabilitation and medicine in general [1]. The realization of the electronics depends on the specific approach and application, although there are a few major common concerns such as wiring, crosstalk or parasitic capacitors. Large tactile sensors with a high number of tactels (a tactel is a single sensing point on a tactile sensor array) and real-time operation are often required. Some pre-processing on the sensory plane results in a reduction of the amount of information to be transmitted to the central decision unit [2–10]. Moreover, detection and processing circuitry should be located near the sensor to avoid problems caused by long wiring runs. It also needs to have a low number of integrated circuits and I/O connections. This reduces the number of cables and allows it to be housed in hands and grippers.

Many tactile sensors have been implemented with technologies that allow the incorporation of circuitry on the same substrate [7–10]. These implementations achieve high spatial resolution, so they are suitable for applications such as Minimally Invasive Surgery (MIS), though they have also been developed for use in other environments such as industry. The circuitry implemented on the same substrate performs the signal conditioning. This is common in capacitive sensors because stray capacitors are a key issue, and amplifiers based on switched capacitors can be realized [8]. Certain preprocessing for sensors able to detect normal and shear forces is also realized on chip, as well as switches to address the array. More complex processing can also be carried out such as that for preprocessing of the tactile image based on convolutions [10]. Nevertheless, most tactile sensor systems or artificial skins are composed of patches that contain integrated circuits on a printed circuit board (PCB). Large areas can be covered with these patches that form a network of smart sensors and communicate with a central processing unit through a serial bus [3,4]. Different devices can be used as the core of the hardware in these patches as discussed in the following paragraphs.

ASICs to act as coprocessors for tactile sensors have been reported [5,6] and general purpose Integrated Circuits (ICs) have also been proposed for that task [11]. They can undertake error reduction, compensate for interference and convert analog-to-digital. It is the best choice in terms of area and power efficiency. Moreover, slippage detection in manipulative tasks with hands or grippers has to be done in the range of 2–4 ms. This means the whole array of force sensors of the tactile sensor (tactels) has to be processed in this time (analog-to-digital conversion plus detection algorithm). The high dynamic performance of ASICs allows the detection of slippage [6]. Unfortunately, ASICs are quite rigid and their programmability is low, so the possibility of updating their functionality once they are fabricated is limited.

Other implementations are based on microcontrollers [2–4]. This strategy usually requires a higher number of devices in the PCB board. This means that a large area is needed because of the space they take up as well as due to a more complex wiring. Furthermore, tactels are read and processed sequentially, hence the response time is poor and slippage detection for a piezoresistive sensor with a high number of tactels is not feasible. However, this approach allows the design to be updated, so the tasks to be carried out by the microcontroller can be changed simply by programming it again. Further improvements of the performance are achieved if the hardware is based on a PSoC. These devices have a set of analog and digital blocks to be configured by the user so hardware is reduced when compared to other standard microcontrollers. Nevertheless, the size of the array that can be addressed depends on the resources implemented on the PSoC and the number of input and output pins. Moreover, though the on-chip blocks allow some level of parallelism in the signal conditioning, programmed algorithms are executed in a sequential way.

The performance of a FPGA falls between these two previous strategies. They are flexible devices because they can be programmed, and at the same time they have a high dynamic performance due to the parallel processing they allow [12]. The main advantage of this strategy is the possibility of performing quite complex pre-processing in real time. As the system becomes more and more complex, many tactile sensors are used, for instance in fingers and palms, so the huge amount of data provided by these sensors should be pre-processed for the main controller to be able to manage it in real time. On the other hand, FPGAs do not commonly have analog-to-digital converters. Therefore, the use of external converters could increase the complexity and cost of the circuitry.

This paper demonstrates an implementation that does not need such external converters. It is based on the direct connection of sensors to microcontrollers [13]. Since the FPGAs have many I/O pins, they allow a very direct connection between the tactile sensor and the device. The smart sensor thus obtained is compact and powerful in terms of real time processing capability. This strategy was proposed by the authors in [14], where an implementation based on active integrators was also proposed to cancel crosstalk and cope with large array signal conditioning. An implementation of this circuitry for a specific raw sensor is presented in this paper. Moreover, signal conditioning circuits based on a PSoC and a microcontroller have also been made for the same sensor for the purpose of analysis and comparison. These circuits are based on ideas previously presented in conferences and journal briefs [15,16]. The comparison is to be taken as an approximation, since final performance depends on many factors, for instance the PCB technology, the encapsulation of integrated circuits and electronic components, or the specific device chosen for the electronics to be based on.

## 2. Piezoresistive Sensors and Crosstalk

**Figure 1.**
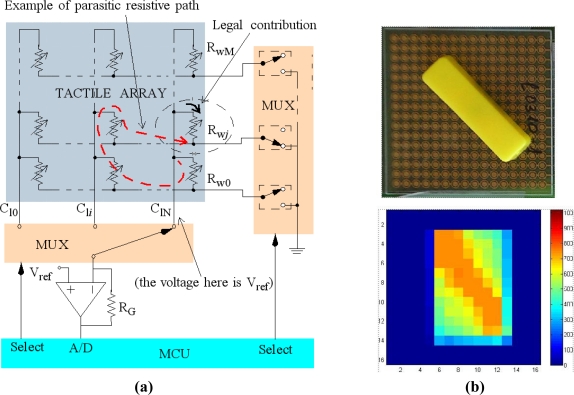
**(a)** Electronics for piezoresistive tactile sensor that does not cancel crosstalk; **(b)** Tactile image obtained by a slash bar on it.

Many tactile sensors are made of sheets of piezoresistive materials. The sheet covers an array of electrodes and we obtain an array of force sensing resistors as tactels. However, if a continuous sheet is used, parasitic resistors are present between tactels in the array. Moreover, crosstalk is present even if the array is composed of discrete force sensors once they are arranged in rows and columns [16,17]. The reason is that the addressing tracks are shared by many tactels and they form resistive paths that cause crosstalk. A circuitry that does not cancel crosstalk is depicted in Figure 1(a), where an example of a parasitic path is also shown. Crosstalk due to the electrical addressing (there can be a certain crosstalk for mechanical reasons) is not registered if a single tactel is pressed. However, if a group of tactels are pressed crosstalk alters the readings for them. It can be hidden if the pressed area presents some symmetry with respect to rows or columns, but it is clear in the other cases. For instance, Figure 1(b) shows the output of a sensor with the signal conditioning in Figure 1(a) for a slash bar on it. Note that there is significant crosstalk. A few strategies to reduce the interference caused by these resistive paths have been proposed [18], although the best one that is commonly implemented is *grounding*. Its goal consists in having the same voltage at both sides of parasitic resistors, so they are virtually short-circuited. Circuits that implement this strategy will be shown in the next sections.

## 3. Electronics Based on a Microcontroller

**Figure 2.**
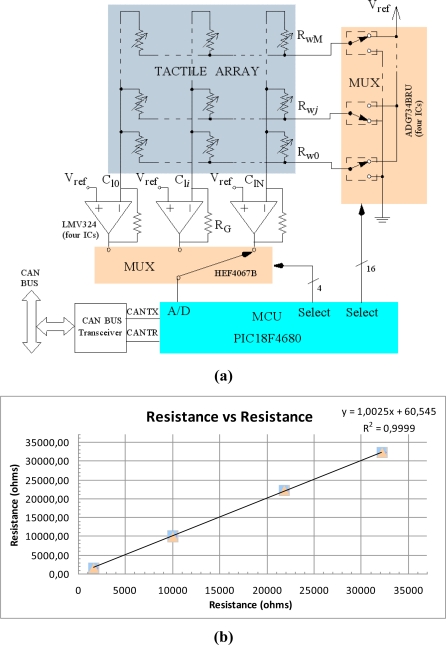
**(a)** Electronics based on Microcontroller; **(b)** Result of the measurement of a few resistors *vs*. its value taken by a multimeter.

The local electronics for a tactile array of *N* columns and *M* rows based on a microcontroller PIC18F4680 (Microchip) are shown at Figure 2(a). These electronics are in charge of scanning the array, storing the data and sending it via CAN bus to a central processing unit. The latter has been a personal computer with a card to communicate with CANOpen for the results of this paper. This figure also illustrates the grounding strategy used to cancel crosstalk. Note that there is one operational amplifier per column in Figure 2(a). Their purpose is to set the voltage *V_ref_* at the tracks of all columns. Since the voltage of all rows that do not contribute to the output is also set to *V_ref_*, any possible parasitic path is short circuited. The output voltage of a column is given by:

(1) $$V_{\mathrm{out}}=(\frac{R_{G}}{R_{\mathrm{ij}}}+1)V_{\mathrm{ref}},\;V_{\mathrm{ref}}\leq V_{\mathrm{out}}\leq V_{\mathrm{DD}}$$

where *R_ij_* is the force dependant resistance of the element *ij* in the array, and *R_G_* is the resistance to set the gain of the transresistance amplifiers at the output of every column. $R_{G}=(\frac{V_{\mathrm{DD}}}{V_{\mathrm{ref}}}-1)R_{\mathrm{ij}\;\text{min}}$ at Figure 2, where *R*_*ij*min_ is the minimum value of *R_ij_* determined by the pressure range of our application. Note that the output range is reduced by V*_ref_* in this implementation. However, the A/D converter in the microcontroller can be configured to take V*_ref_* as low voltage and still provide 10 bits if the excursion is higher than 3 volts. The operational amplifiers were chosen to be rail to rail to accomplish this, and the output can rise up to V_DD_ −40 mV@5 V@25 °C typically. The range in resistance is therefore:

(2) $$\frac{R_{G}V_{\mathrm{ref}}}{V_{\mathrm{DD}}-V_{\mathrm{ref}}}\leq R_{\mathrm{ij}}\leq \frac{R_{G}V_{\mathrm{ref}}}{V_{\mathrm{DD}}-V_{\mathrm{ref}}}2^{\mathrm{NOB}}$$

where *NOB* means number of bits.

The operational amplifiers should provide enough current; the maximum demanded current being V*_ref_*/*R*_*ij*min_. The sourcing current of the LMV324 (Texas Instruments) is typically 80 mA for 5 V of supply voltage. Moreover, current switches in the analog multiplexor ADG734 (Analog Devices) must withstand a current *N* × V*_ref_*/*R*_*ij*min_ and perform as very low resistances when they are ON. Their typical resistance value is 2.5 Ω so the error introduced is very low. Another source of error is the noise in V*_ref_*. The voltage reference REF3012 (Texas Instruments) with 0.2% accuracy is used to reduce this error. Note also that V*_ref_* is a reference, not a voltage supply, since it does not provide any current in static condition, so the error it causes is negligible once the transitory is concluded. The input offset of the Op-Amp (operational amplifier) is very small, only 1.7 mV typically, so the error it causes is also small. Figure 2(b) shows the measurement of a few resistors obtained from the electronics in Figure 2(a) *versus* the value registered by a multimeter. Thirty samples were taken for every resistor value. If the electronics is designed to be connected to a CAN bus, a regulator is required to provide the low voltages for the microcontroller. The regulator should be able to provide a current of (V*_ref_*/*R*_*ij*min_) × *N* plus the current demanded by the microcontroller and the other integrated circuits. A TL750L05 (Texas Instruments) was chosen for the results of this paper. All the integrated circuits were low power to reduce power consumption. Finally, the MCP2551 (Microchip) CAN bus transceiver was used to implement the interface to CAN bus. Please find performance data for this implementation in Section 7 and Table 1.

## 4. Electronics Based on a PSoC

**Figure 3.**
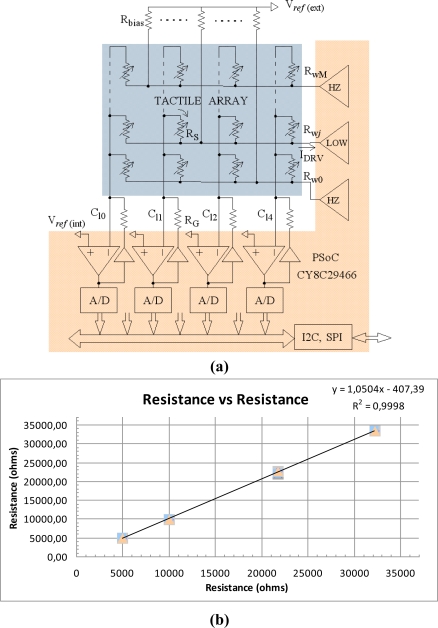
**(a)** Electronics based on PSoC; **(b)** Result of the measurement of a few resistors *vs*. its vaule taken by a multimeter.

A more compact implementation can be achieved if the electronics is based on a Programmable System on Chip (PSoC) instead of a standard microcontroller. These devices implement more analog blocks that can be used in the acquisition circuitry. For instance, operational amplifiers in Figure 3(a) are implemented on-chip. The architecture in Figure 3(a) is similar to that in Figure 2 and Equation (1) is valid. Nevertheless, due to the limitations in on-chip resources some differences are observed. First, just four columns can be implemented. Second, rows are addressed directly by output pins of the PSoC whose drivers can only provide the voltage levels related to logical high and low values. The output that is set at logical low level drives the active row and its corresponding pin [R_w*j*_ in Figure 3(a)] sinks the current *I_DRV_*. The remaining output pins are set at high impedance. In this way it is possible to implement grounding if a few bias resistors and an auxiliary voltage reference are used. They are R_bias_ and V*_ref_* in Figure 3. Note that this bias forces a voltage V*_ref_* at rows that are not driven and parasitic paths are short circuited because columns are also set to V*_ref_* due to the negative feedback in the amplifiers. As in Figure 2, limited gain and offset of these amplifiers are sources of error. Typical input offset voltage is as low as 1.3 mV for the PSoC of this paper (Cypress CY8C29466), so the error it introduces is negligible. The amplifiers also have to provide the current sunk by the external circuitry. There is an analog buffer between the Op-Amp output and the resistance R_G_ in the feedback loop. This buffer is able to provide up to 40 mA.

V*_ref_* at non inverting input of the Op-Amps is generated internally because of the lack of I/O pins to take this reference from an external source or to share it with the biasing resistor network. Therefore, an extra voltage source is required to provide the intermediate voltage (V*_ref_* = 2.5 V). The latter can be obtained with a voltage regulator. Another alternative is the use of the reference V*_ref_* generated on-chip, although one pin is dedicated to providing access to it and the number of columns is reduced by one (three with the PSoC of this paper). The mismatching between voltage levels of biasing sources is another source of error. The external reference is generated with the regulator LP2985 (Texas Instruments) for the results of this paper. Another important equation is:

(3) $$V_{\mathrm{ref}}\;(\frac{1}{R_{\mathrm{bias}}}+\frac{4}{R_{S\;\text{min}}})\leq I_{\mathrm{DRV}\text{max}}$$

to guarantee that the current sunk by the row driver does not exceed the limit imposed by the PSoC (*I*_*DRV*max_). For the device of this paper this current is 25 mA. The output impedance of the row driver introduces error too, similarly to the ON resistance of analog switches at Figure 2. The maximum voltage at this output for 25 mA is 0.75 V, so a linear approximation results in around 30 Ω of output impedance. Figure 3(b) shows the measurement of a few resistors obtained from the electronics in Figure 3(a) *versus* the value registered by a multimeter. Thirty samples were taken for every resistor value.

The operational amplifiers are rail to rail, which improves the dynamic range. However, it is important to say that this range is half that of the converters, because the range of the input is from V_DD_/2 to V_DD_. Therefore, Equation (2) is valid but *NOB* is one bit less than the resolution of the A/D converter on the PSoC. Finally, the PSoC implements resources to ease communication through serial bus (Universal Asynchronous Receiver-Transmitter, I2C and SPI). Please find performance data for this implementation in Section 7 and Table 1.

## 5. Electronics Based on an FPGA

### 5.1. Direct Connection with Passive Integrators

**Figure 4.**
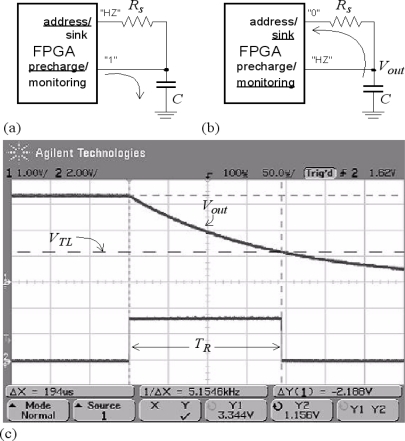
Direct connection with passive integrators.

The following procedure describes how to connect a resistive sensor to a device with digital interface, *i.e*., a device designed to interface with digital signals. A thorough study of this strategy for resistive sensor-to-microcontroller interfaces is reported in [13]. The approach is illustrated in Figure 4. In a first phase the capacitor is charged through the pin named “precharge/monitoring” at Figure 4(a). In the second phase it is discharged. To do that, the pin “address/sink” at Figure 4 is set to a digital low value and it sinks the current from the capacitor. A timer starts its count at this instant, and the voltage across the capacitor is monitored by the input with label “precharge/monitoring” at Figure 4(b). When it takes a value V_TL_ corresponding to a digital “0”, the count stops. The measured time is:

(4) $$T_{R}=R_{S}C\;\text{ln}\;(V_{\mathrm{DD}}/V_{\mathrm{TL}})$$

where V_DD_ is the voltage across the capacitor at the beginning of the discharging phase. Note that the resistance can be obtained from Equation (4) once *T*_R_ is measured. Figure 4(c) shows the voltage across the capacitor and the trigger signal to stop the count in the discharging phase measured with the scope for a given resistance value.

Regarding pre-processing of tactile data on local electronics, slippage detection is the task with the highest dynamic requirements. Specifically, it is detected at frequencies around 250 Hz [6]. Therefore, we should be able to carry out the A/D conversion of a whole array in the range of 2–4 ms. Since we can perform many A/D conversions in parallel, this does not mean we should read the array in 4 ms/(*M* × *N*) where *M* × *N* is the number of tactels. Instead, we have to read the array in 4 ms/*N*, where *N* is the number of columns in the array, as the next section describes. This means the time constant is very small and trigger noise effects are negligible, so the resolution of the time to digital conversion is given by [13]:

(5) $$\mathrm{ENOB}\approx \mathrm{lb}\left\{f_{\mathrm{CLK}}C\;\text{ln}\;(V_{\mathrm{DD}}/V_{\mathrm{TL}}){\Delta R}_{S}\right\}$$

where *ENOB* means Effective Number Of Bits, *lb* is the binary logarithm, and Δ*R*_S_ is the range of the resistance read from the tactile sensor. For a given Δ*R*_S_ and a required resolution we determine the value of *C* and *f_CLK_*.

### 5.2. Interface for Low-Medium Size Arrays

**Figure 5.**
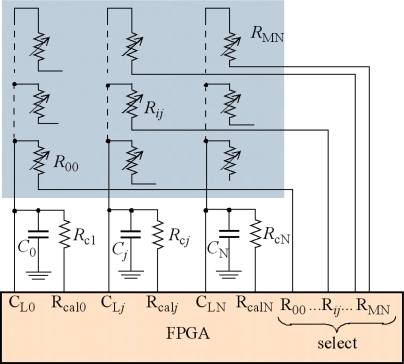
Direct interface tactile sensor-FPGA for low-medium size arrays.

Figure 5 shows a direct interface to a tactile sensor. The high number of I/O pins of the device is exploited to address the tactels. The array is read as follows. First, the capacitors *C*_0_...*C_j_*...*C*_N_ are pre-charged by setting pins C_L0_...C_L*j*_...C_LN_ to ‘1’ and the remaining I/O pins to HZ. Then, a whole row is selected by setting its corresponding I/O pins to ‘0’. For instance, pins R_*i*0_...R*_ij_*...R_*i*N_ are set to ‘0’ while the remaining “select” I/O pins are set to HZ. The capacitors are discharged and the voltages across them are monitored by pins C_L0_...C_L*j*_...C_LN_, which are set to HZ. A set of timers are started in the FPGA at the beginning of the discharging phase and their counts are stopped when the low threshold V_TL_ is reached at the related column pins. Therefore, a whole row is read in parallel. Note that there is a dedicated pin per tactel in the array. It is not possible to address a whole row with a pin only because the tactels become connected to each other and the charge in the capacitors is redistributed among them by many different resistive paths. An implementation with isolated tactels is possible, for instance [19] reports a sensor with 272 tactels that are addressed with a track per tactel plus a common electrode.

To obtain the resistance *R_ij_* from Equation (4) we have to know the values of *C*, V_DD_ and V_TL_. However, we ignore their exact value, and they can drift with time, power voltage supply or temperature. A calibration procedure can be implemented to compensate such lack of knowledge and/or interferences. The simplest consists in using calibration resistors, like those labeled *R*_c1_,...*R*_c*j*_...*R*_cN_ at Figure 5. The whole set of calibration resistors is read as a row of the tactile array. Then the resistance after calibration is computed as:

(6) $$R_{\mathrm{ij}}=(t_{\mathrm{Dj}}/t_{\mathrm{Dcj}})R_{\mathrm{cj}}$$

where *t_Dj_* and *t_Dcj_* are the contents of the timers in the FPGA corresponding to the count of the discharging times for the resistor R*_ij_* and the calibration resistor R*_cj_* respectively. A two-point calibration is better but it requires another set of resistors for calibration. The calibration procedure also takes time, however we can do it once per tactile image frame, or even at a lower rate to increase the dynamic performance.

As in the case of the other circuitry described above, the impedance associated to the pins R_00_,…R*_ij_*,…R*_MN_* in the FPGA introduces an error. This impedance is in series with the resistance R*_ij_* that is being measured so it is added to the result of the measurement in the simplest case. We can consider this impedance approximately constant because it is associated to the channel of an NMOS transistor working in the linear region. If we use the calibration procedure the error is minimum for resistances close to the calibration one. Another source of error is the finite impedance of the inputs C_L0_,…C_L*j*_,…C_L*N*_ when they are set to HZ to monitor the voltage drop in the capacitor. A leakage current in the order or 10uA is present at this input.

The total number of I/O pins in Figure 5 dedicated to address the tactile sensor is *M* × *N* + 2 × *N*. This limits the size of the array that can be addressed in this way. For instance, an array of 8 × 8 tactels requires 96 pins of the FPGA to implement its interface. To obtain the results of this paper we have used a Spartan 3AN-50 (Xilinx) with 108 I/O pins, thus it is possible to implement this strategy. Its main advantage is that the number of integrated circuits is only one, so the interface is very compact. However, there are a high number of tracks and pins to connect and the PCB is more complex.

### 5.3. Interface for Large Arrays

**Figure 6.**
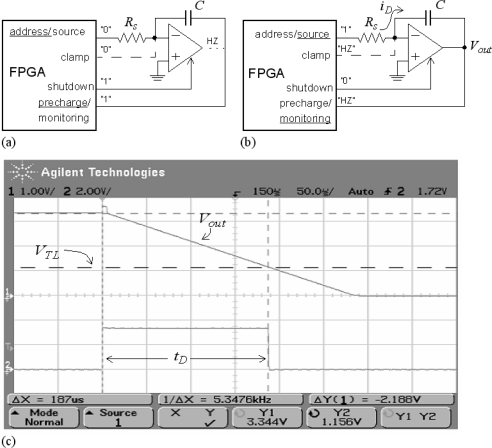
Direct connection with active integrators.

If the tactile array has a high number of tactels, the strategy in Figure 5 is not feasible. For instance, up to 288 pins are required to implement the interface with an array of 16 × 16 tactels. We propose the use of active instead of passive integrators to implement the direct connection for this case. Figure 6 shows the implementation for a single resistive sensor. The concept is the same as for the use of passive integrators, in Figure 6(a) the capacitor is charged, and it is discharged in Figure 6(b) with a constant current given by *i_D_* = *V_DD_*/*R_S_* (note the linear discharge at Figure 6(c)). Nevertheless, there are some differences. First, we need to ‘turn off’ the operational amplifier in the charging phase of the capacitor because otherwise its output interferes in the charging and it is not completed. So we need an amplifier with ‘shutdown’ input and a dedicated pin of the FPGA to address it. Second, another output of the FPGA is devoted to clamp the non inverting input of the amplifier to a voltage close to ground in the charging phase. The charge would be completed without this clamp but the time to charge the capacitor would depend on the value of the resistor. Therefore, to reduce the time for the analog to digital conversion the use of this clamp is recommended. However, it can be removed in the case of low dynamic requirements to reduce the number of pins of the FPGA dedicated to the analog to digital conversion.

**Figure 7.**
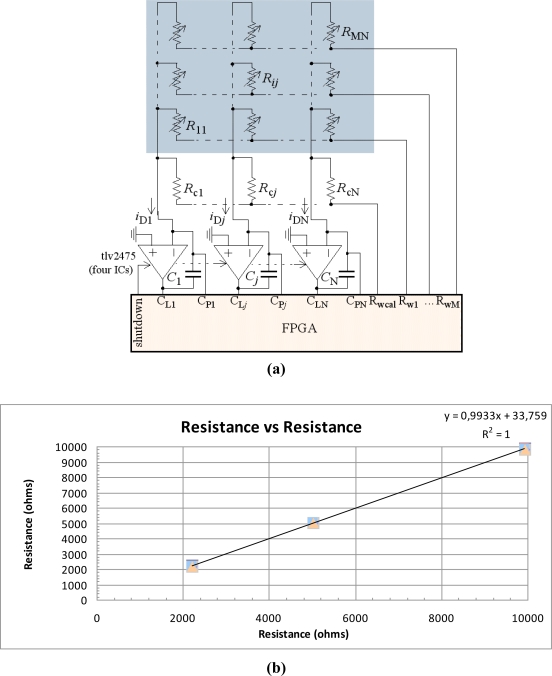
**(a)** Direct interface tactile sensor-FPGA for large arrays; **(b)** Results of the measurement of a few resistors *vs*. its value taken by a multimeter.

Figure 7 shows the proposal to implement the interface with the tactile sensor in this case. Passive integrators are replaced by active ones and we obtain a circuitry with meaningful similarities to Figures 2(a) or 3(a). Note that the pin to shutdown the amplifiers is shared by them. Note also that columns in the array are virtually grounded due to the negative feedback loop implemented by the active integrators. This means we can follow the usual strategy in Figures 2(a) and 3(a) to short circuit the resistors that are not selected and avoid that they contribute with parasitic currents to the output. This can be done as follows. In a first phase, the selection pins R_w0_...R_w*i*_...R_wM_ are set to ‘0’. The tactile array and the FPGA share the ground, therefore a ‘0’ at these pins means this voltage is almost 0 and the resistors of the whole array are short circuited. At the same time pins C_L0_...C_L*j*_...C_LN_ are set to ‘1’ (voltage V_DD_), C_P0_...C_P*j*_...C_PN_ are set to ‘0’, shutdown is set to ‘1’ and the capacitors C_0_...C*_j_*...C_N_ are charged to a voltage V_DD_ across them. In the second phase, the set of column timers start their counts, and a row is selected. For instance R_w*i*_ is selected and there is a voltage drop V_DD_ across tactels R_*i*0_...R*_ij_*...R_*i*N_. The amplifiers are turned on by setting shutdown to ‘0’. Pins C_L0_...C_L*j*_...C_LN_ and C_P0_...C_P*j*_...C_PN_ are now at HZ. Therefore, currents *i_Dj_* = *V_DD_*/*R_ij_* where *j* = 1…*N* flow into the integrators, and the voltages at C_L0_...C_L*j*_...C_LN_ decrease. They decrease until threshold V_TL_ is reached at every pin C_L*j*_, then the count of the corresponding timer stops. At this time C_P*j*_ is set to ‘0’ to avoid that the voltage at the inverting input of the amplifier grows and generates interferences in tactels of the same row. We can obtain the value of the resistance from:

(7) $$R_{\mathrm{ij}}=\frac{V_{\mathrm{DD}}}{(V_{\mathrm{DD}}-V_{\mathrm{TL}})C_{j}}t_{\mathrm{Dj}}$$

where *t_Dj_* is the time measured by the timer. If this time is short enough, *i.e*., the time constant is small enough, we can neglect the trigger noise at threshold V_TL_ and take into account only quantization noise to obtain the resolution given by:

(8) $$\mathrm{ENOB}\approx \mathrm{lb}\left\{f_{\mathrm{CLK}}C_{j}(\frac{V_{\mathrm{DD}}-V_{\mathrm{TL}}}{V_{\mathrm{DD}}}){\Delta R}_{\mathrm{ij}}\right\}$$

Note that a high current flows now from pin R_w*i*_. A current up to 24 mA can be sourced by these pins for the device of the prototype of this paper. This current is sunk by the output of the Op-Amp, so it must be chosen to accomplish this. Note also that its output must be rail to rail if the supply voltage is the same as for the FPGA. The TLV2475 (Texas Instruments) is able to sink 10 mA at 180 mV off the rail. The limit of 24 mA in sourcing current from the pins that select the active row imposes the lower limit of the resistance that can be measured which is *R*_*ij*min_ = *N* × *V_DD_*/24 mA. For a target *ENOB* the upper limit in the range of the resistances that can be measured is derived from Equation (8). Note that there is a tradeoff here with the conversion time since the larger the value of R*_ij_* is, the larger the discharging time *t_Dj_* in Equation (7). It is possible to reduce the discharging time while preserving the resolution given by Equation (8) if a smaller capacitance is chosen for C*_j_* (note that the result of Equation (8) is the same because ΔR*_ij_* has increased). Nevertheless, the time constant is reduced in this way for small resistor values and the approximation of neglecting the trigger noise is less valid. Figure 7(b) shows the result of measuring a few resistors with the electronics in Figure 7(a) *versus* the data from a multimeter. The data was obtained by *f_CLK_* = 50 MHz, C*_j_* = 50 nF, R*_ijmin_* = 2.2 KΩ and R*_ijmax_* = 9.927 KΩ. The time invested to scan the 16 × 16 array is 5.5 ms. The standard deviation for the resistor of 2.2 KΩ is 1.18 Ω, so the approximation of neglecting the trigger noise is valid.

Similarly to the case with passive integrators, the impedance of the pin R_w*i*_ introduces an error, although this pin is sourcing the current now and it is set to a logical high value, so the parasitic resistor is that associated to the channel of the high side MOS transistor in the output buffer. It can be considered constant if the transistor works in linear region. This resistance depends on the specific output buffer that is chosen but it is around 25 Ω for 16 mA and up to 37.5 Ω for 25 mA (estimated from values of V_OH_ and I_OH_ provided in the datasheet). Performance data for the implementations described above may be found in the following section and Table 1.

## 7. Discussion

**Figure 8.**
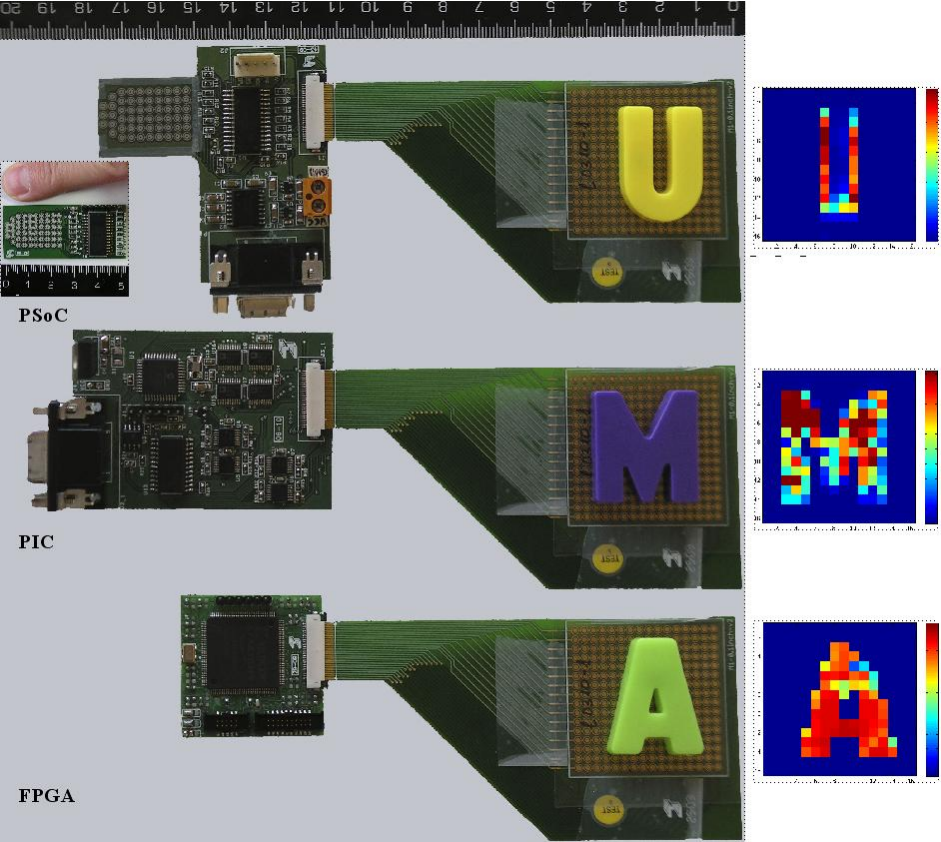
Photograph of the electronics of the prototypes (right) and tactile images obtained from them (left).

Figure 8 shows photographs of prototypes of electronics based on Microcontroller, PSoC and FPGA. They are connected to the same piezoresistive tactile sensor [20], although the PSoC is not able to scan the whole array (the detail in the figure corresponding to the PSoC shows an implementation where the PSoC is the only IC on the PCB and the electrodes are arranged to resemble the shape of a fingertip). The output of these sensors for different letter-shaped profiles on them is shown on the left of Figure 8. Moreover, Table 1 presents results obtained with these electronics, where the transceivers are not counted in the number of ICs required. As mentioned in the introduction, the comparison should be made with care because different alternatives could be followed in every case. Nevertheless, it provides interesting information for hardware designers.

Table 1 also includes data for another two proposals. First, the circuitry in Figure 2(a) was designed in a straightforward way and an analog multiplexor was chosen to drive the rows. However, it is possible to implement the strategy in Figure 3(a) and replace the multiplexor with an array of resistors. This would save four ICs and some area and power consumption. However, the inputs sink up to 8.5 mA for a voltage drop V_OL_ = 0.6 V. This means that there will be larger limitations in the range of resistance that can be measured and the errors due to the output impedance will be larger than those in the implementation of Figure 2(a). Since the PIC18F4680 has 11 A/D channels, it is also possible to take off the multiplexor MC14067 if the number of columns to address is lower than eleven. Note that this alternative reduces the number of integrated circuits to five without the transceiver. Second, the implementation in Section 5.2 was tested with a general purpose development card. Some data such as power or area consumption cannot be provided for this reason but other meaningful data is worth including in the table. The data marked with an asterisk are estimations from the datasheet.

The implementation based on the PIC has the best performance in resolution and power consumption. It is also able to scan quite large arrays of 16 × 16 tactels, although it is the less compact. The implementation based on the PSoC is very compact due to the resources implemented on chip and to the high driving capability of the I/O pins. The CY8C29446 has 12 configurable analog blocks. Many functions can be implemented with these blocks and are available as user modules like Delta-Sigma, Successive Approximation or Incremental Analog to Digital converters and programmable amplifiers. Moreover, the blocks can be configured by the designer if there is not a module in the library, which was the case in Figure 3(a). The dynamic performance can be improved if more than one converter is implemented to work in parallel. This is especially true for low resolution converters. For instance, the 14 × 4 array is scanned in 1.36 ms if four 6 bit successive approximation converters are used (the effective resolution is 5 bits, as explained in Section 4). The power consumption shown in Table 1 was measured when low value resistors of 1.68 KΩ where connected at the output to simulate the access to a row whose tactels have a high pressure on them. Note that the power consumption of the PSoC is similar to that shown by the PIC in absolute terms. However, the implementation based on PSoC consumes approximately five times per tactel more than the implementation based on PIC. This is due in part to the fact that V*_ref_* is 2.5 V in Figure 3(a) while it is 1.3 V in Figure 2(a). The maximum resolution achieved is 8 bits to scan the 14 × 4 array in 31.8 ms, while the PIC scans 16 × 16 tactels in 15.6 ms with 10 bits of resolution.

Although a few tradeoffs between input range, resolution and speed are present, the implementation based on direct connection to an FPGA is the fastest. It was designed for a resolution of eight bits and an input range up to 8 KΩ. Larger input ranges can be achieved preserving the dynamic requirements with smaller capacitors, but effective resolution could be less for low resistance values corresponding to high pressures. The FPGA is more expensive than the PIC and the PSOC, and the PCB has four layers. The implementation with active integrators needs five ICs, a number higher that for those based on PSoC or on direct connection with passive integrators, but lower than for the implementation with the PIC. The power consumption per tactel is considerably lower than that with the PSoC and twice that obtained with the PIC. It also requires a four layer PCB. It is particularly interesting to note the possibility of implementing concurrency on the FPGA. This allows a very high dynamic performance to be obtained and algorithms with high requirements in terms of speed, such as slippage detection, can be dealt with [6,21].

Another test was carried out to examine the performance of the implemented circuits in the crosstalk cancelation. A resistor of high value was surrounded by resistors of low value in an array with shared tracks to address rows and columns that model a tactile sensor. This was considered preferable to making it with a tactile sensor to isolate crosstalk caused by electrical and mechanical sources. Crosstalk was not observed in any case, it was smaller than a Least Significant Bit. However, crosstalk was very significant if the electronics in Figure 1(a) was used instead of the others.

Finally, despite the circuitry being designed to minimize errors, it is worth highlighting here that many raw piezoresistive tactile sensors based on conductive rubbers or polymers show non linearity, hysteresis, drift and poor repeatability [20]. The powerful electronics based on FPGAs provides hardware able to carry out complex computations in a short time and effectively compensate these errors as much as possible. Moreover, despite the fact that the intelligence of the sensor can cancel many errors, the effective resolution of the resulting sensor will be less than that predicted without taking into account the limitations of the raw sensor. This could justify the choice of a circuitry with an analog to digital converter of lower resolution. However, another significant issue has to be observed. It is the nonlinear relationship between the force and the resistance the raw sensor shows. Moreover, note that the relationship between the output voltage (the input of the A/D converter) and the resistance R*_ij_* is not linear in Equation (1). This nonlinearity can compensate that imposed by the raw sensor between force and resistance, but we will have different responses depending on the specific implementation [20]. Nevertheless, the non linear behaviour between the measured (the pressure) and the input of the A/D converter has to be taken into account to evaluate the impact of the limited resolution of the A/D conversion.

## 8. Conclusions

Some general conclusions may be drawn from the data obtained for the three discussed approaches, although they need to be qualified by other aspects as done in the previous section. The shortest scanning time is obtained by the implementation based on the FPGA, while that based on the PSoC obtains the longest and the PIC-based one gives a time that is in between and provides the highest resolution in number of bits. Nevertheless, very short scanning times can be achieved with the PSoC for low resolutions. Power consumption per tactel is also greater for the PSoC-based implementation than for the others, and the PIC-based implementation consumes the least. However, the most compact realization is achieved by the PSoC, although a smaller number of tactels can be addressed. The implementation based on FPGAs is also quite compact but it needs four-layer PCBs. This fact, together with the price of the main integrated circuit, makes the cost of the latter higher than the hardware based on the PIC or the PSoC. Finally, it is worth mentioning that the implementation based on FPGAs has a high degree of parallelism in the proposed analog-to-digital conversion and in the algorithms running on it, which is a key issue in real time operation.

## Figures and Tables

**Table 1.** Comparison of Different Implementations.

**Hardware based on**	**Complexity**			**Scanning Time(ms)@Nr of bits of A/D conversion**		**Average Power Consumption with/without reading a row**		**Cost of the main IC ($)**	**Hardware Resources**
	**Nr. of ICs**	**Nr. of PCB layers**	**Size of the array *M* × *N***	**Absolute**	**Relative to array size**	**Absolute**	**Relative to array size**		
PIC18F4680 (L)	10	2	16 × 16 (256)	15.6 (10 b)	0.975/row	37 mA@5 V/25 mA@5 V	0.72 mW/tactel	6.32	CPU 18PIC@40 MHz, 3.3 Kb SRAM, 64 Kb FLASH, 1 8 × 8 Multiplier ALU, 36 digital I/O, 8.5 mA@I_OL_ 0.6 V@V_OL_, 11 input analog A/D channels, Up to 10 bits A/D converter, UART, SPI, I2C, CAN facilities
PIC18F4680 (M)	5	2	11 × 19 (201)	10* (10b)	0.515*/row	NA	NA		
PSoC CY8C29466	1	2	14 × 4 (56)	31.8 (8 b) 7.2 (7 b) 1.36 (5 b)	2.27/row (8b) 0.51/row (7b) 0.10/row(5b)	37.7 mA@5 V/20 mA @5 V	3.36 mW/tactel	8.02	CPU Core M8C@24 MHz, 2 Kb SRAM, 32 Kb FLASH, 2 8 × 8 Multiplier ALU 24 digital I/O, 12 Analog I @24 mA sink, 4 Analog O @30 mA 12 analog blocks, 8 digital blocks Up to 14 bits A/D converters UART, SPI, I2C facilities
FPGA PI SPARTAN 3AN-50	1	4	8 × 8 (64)	4*(8b)	0.5*/row	NA	NA	8.94	50 MHz, 50 K System Gates, 176 Configurable Logic Blocks, 3 18 × 18 dedicated multipliers, 11 Kbits Distributed RAM, 54 Kbits Block RAM, 1 Mbit FLASH, 108 I/O pins @ 24 mA I_OH_ V_OH_
FPGA AI SPARTAN 3AN-50	5	4	16 × 16 (256)	5.5 (8b)	0.343/row	100 mA@3.3 V/60 mA@3.3 V	1.3 mW/tactel		
